# The Effect of Feeding with Chironomid and *Artemia* on Fatty Acids and Amino Acids Profiles in Persian Sturgeon (*Acipenser persicus*) Larvae

**DOI:** 10.1155/2024/6975546

**Published:** 2024-05-13

**Authors:** Iraj Efatpanah, Bahram Falahatkar, Mir Masoud Sajjadi, Maryam Monsef Shokri

**Affiliations:** ^1^Fisheries Department, Faculty of Natural Resources, University of Guilan, Sowmeh Sara, Guilan, Iran; ^2^Department of Marine Sciences, The Caspian Sea Basin Research Center, University of Guilan, Rasht, Guilan, Iran; ^3^International Sturgeon Research Institute, Iranian Fisheries Science Research Institute, Agricultural Research, Education and Extension Organization (AREEO), Rasht, Iran

## Abstract

This study aimed to examine the effect of various live foods on the fatty acids (FAs) and amino acids (AAs) profiles in Persian sturgeon (*Acipenser persicus*) larvae. One thousand and two hundred larvae were cultured in circular concrete tanks, and four treatments were administered as: (1) *Artemia* + *Daphnia*, (2) *Artemia*, (3) *Artemia* + Chironomid, and (4) Chironomid. Each treatment was considered as three replicates over an 11-day period. At the end of the experiment, treatment 1 (*Artemia* + *Daphnia*) showed the highest average weight of larvae, and the lowest weight was observed in treatment 4 (Chironomid). Survival rate ranged from 83.84% to 88.86% and no significant difference was observed among the groups (*P*  > 0.05). Among *Artemia*-fed larvae, the predominant FAs were docosahexaenoic acid (DHA), oleic acid (*ω*9), and monounsaturated fatty acids (MUFA), while saturated fatty acids (SFA) and polyunsaturated fatty acids (PUFA) (*ω*3 + *ω*6) were present in a lesser proportion (*P*  < 0.05). In larvae fed with *Artemia* and *Daphnia*, the predominant proportions were observed in SFAs, eicosapentaenoic acid (EPA), *ω*3, DHA + EPA, and the n3/n6 ratio, all registering the highest percentages. Conversely, MUFA, *ω*6, and the DHA/EPA ratio displayed the lowest percentages (*P*  < 0.05). Moreover, larvae fed with *Artemia* exhibited higher levels of *ω*6, PUFA (*ω*3 + *ω*6), and DHA/EPA ratio. In contrast, larvae fed with Chironomid showed lower levels of EPA, DHA + EPA, and n3/n6 ratio (*P*  < 0.05). Among larvae fed with Chironomid, solely the DHA/EPA ratio exhibited a higher value compared to larvae fed with *Artemia* and *Daphnia* (*P*  < 0.05). The amount of leucine in fish fed *Artemia* + *Daphnia* was more than the other treatments (*P*  < 0.05). This study revealed a significant difference in amino acids composition among various live foods (*P*  < 0.05), but no significant difference in AAs was observed in the body of Persian sturgeon larvae (*P*  > 0.05). The results of this study suggest that the Persian sturgeon larvae possess the ability to maintain a balanced state of AAs. It is also evident that the FA profile of different live foods can affect the overall FA levels in the body of Persian sturgeon larvae, ultimately contributing to the enhancement of fish survival rate and growth.

## 1. Introduction

Persian sturgeon (*Acipenser persicus*) is one of the five valuable sturgeon species in the south of the Caspian Sea [1, 2]. Given that sturgeon larvae are unable to feed on formulated dry feed, the practice of utilizing live foods becomes essential in hatchery setting. The use of live foods containing beneficial compounds conducive to the growth and survival of fish larvae, and that compatible with their digestive system, can significantly enhance the efficiency of the rearing process. The efficiency of live prey depends on providing sufficient amounts of amino acids (AAs) and unsaturated fatty acids (UFAs) for the growth and development of larvae [3, 4].

Due to their small size at hatching and high nutritional quality, *Artemia* nauplii serve as an initial food for many fish species [4, 5]. *Artemia* is recognized as a unique live food in the aquaculture industry, but it is deficient in essential eicosapentaenoic acid (EPA) and docosahexaenoic acid (DHA) [6, 7]. *Daphnia* is a commonly employed live food during the initial days of feeding to stimulate the normal growth and development of digestive system in sturgeon larvae within hatchery environments [1, 8]. Chironomid larvae, also known as blood worms, are widely recognized as a valuable live food source. Their high nutritional value, characterized by high protein content and the presence of essential amino acids (EAAs) [9, 10], renders them suitable as a nutritious food for various fish species, including sturgeons [9].

The fatty acids (FAs) profile of fish throughout different life stages can be influenced by the composition of the consumed food [11, 12]. EPA and DHA are considered essential fatty acids (EFAs) crucial for the growth and survival of the majority of marine fish larvae [4–13]. DHA and, particularly, the DHA/EPA ratio play a significant role in enhancing the growth and survival of fish larvae [14].

The FA composition of the body serves as an indicator of the FA required by the larvae [15, 16]. In general, the EFAs profile of fish carcasses is deemed a reliable indicator for fulfilling the nutritional requirements of larvae [17, 18]. Furthermore, analyzing the AAs profile of larvae is useful for assessing potential dietary AA deficiencies and determining potential imbalances in their diet [18, 19].

The development of Persian sturgeon rearing necessitates sufficient information regarding nutritional requirements to enhance growth and survival. Consequently, this study aimed to investigate the impact of *Artemia*, Chironomid, and *Daphnia* on the AA and FA profiles, as well as the limiting AA of Persian sturgeon larvae. The objective was to establish the correlation coefficient between the EAAs composition of larvae and their respective food sources. The obtained information appears to be instrumental in formulating a suitable diet for the rearing of Persian sturgeon, facilitating their adaptation to dry food in the later stages of development.

## 2. Materials and Methods

### 2.1. Fish and Rearing Conditions

This study was carried out in the larval rearing facilities at the Dr. Yousefpour Marine Fishes Restocking and Genetic Conservation Center (Siahkal, Guilan, Iran). This study utilized 12 circular concrete tanks, each with a diameter of 1.85 m and a depth of 0.5 m, providing a water volume of 810 L per tank. The study was conducted from April to May under the 14 L : 10 D photoperiod. Water, sourced from the Khararoud River, flowed into the tanks at a rate of 20.0 ± 0.2 L/min. The water temperature remained at 17.7 ± 0.7°C, and dissolved oxygen levels were consistently measured at 7.6 ± 0.4 mg/L throughout the experiment. To prevent against the ingress of eggs or larvae from other living organisms, a filter with a mesh size of 100 *µ*m was installed on the inlet pipes for each tank. Persian sturgeon larvae were supplied from fertilized eggs obtained from a wild female and two males captured from the wild stock of the Caspian Sea. Prior to transferring the larvae, the tanks underwent a thorough cleaning and disinfection process using Halamide (Basir Shimi Company, Tehran, Iran) with 60 mg/L for 30 min [20]. For each treatment, three tanks were randomly selected. Subsequently, 1,200 larvae were randomly distributed into each tank, 2 days prior to the start of active feeding.

### 2.2. Experimental Treatments

The larvae were subjected to four treatments over an 11-day period including: (1) *Artemia* + *Daphnia*, (2) *Artemia*, (3) *Artemia* + Chironomid, and (4) Chironomid, with three replications. Live foods were introduced for feeding in 12 days posthatched larvae. In the case of Persian sturgeon larvae, the initial diet comprised *Artemia* nauplii for the initial 3 days, followed by *Daphnia* for the subsequent 8 days. In this study, during the first 3 days, larvae in treatments 1, 2, and 3 were fed with *Artemia* nauplii (Instar 1 with 400–500 micron in size) (Iran Artemia, Tehran, Iran), while Chironomid larvae (Mahiran, Tehran, Iran) were used as the primary feed for the larvae in treatment 4. Feeding was performed 12 times daily at 2 hr intervals, and dead larvae were removed each morning during tank cleaning and siphoning. The quantity of *Artemia* or Chironomid provided for larval feeding was adjusted to 60% of the living biomass in each tank [1]. Beginning 3 days after the initial feeding with *Artemia* nauplii in treatments 1, 2, and 3, and with Chironomid in treatment 4, a shift in diet occurred. From the fourth day onward, *Daphnia* was introduced for feeding in treatment 1, *Artemia* nauplii continued in treatment 2, and Chironomid larvae were supplied for treatments 3 and 4. Following 3 days of this feeding regime, the exact amount of larval feed was determined by measuring the average weight of fish in each tank. Daily maintenance included siphoning out food debris and removing it in the morning, with a count of the dead larvae.

To determine the daily dietary amounts of the larvae during the rearing period, biometric measurements of the fish were performed, determining both the average weight and total biomass using a digital scale with an accuracy of 0.01 g. To facilitate better larval access to food, minimize energy expenditure in capturing prey, and enhance feeding efficiency, the water level in the tanks was reduced by half. Chironomids (*Chironomus plumosus*) (Mahiran Company, Tehran, Iran) were distributed in smaller sizes along the sides of the experimental tanks. Moreover, on the 11th day, larvae from different treatments were sampled to measure the profiles of FAs and AAs.

Artemia cysts (*A. franciscana*) were obtained from Iran Artemia (Tehran, Iran). The standard method [21] for *Artemia* nauplii production involved different steps, encompassing hydration, decapsulation, and hatching. Briefly, cysts underwent decapsulation using a 5% sodium hypochlorite solution at a rate of 15 mL/g of cyst. Subsequently, the decapsulated cysts were hatched in a 100 L Zoug container at a temperature ranging between 28 and 30°C, with a salinity level of 30 ppt. The nauplii were successfully hatched within 18–24 hr, and these actively hatched *Artemia* nauplii were used for the present study.

*Daphnia* were collected from earthen ponds, filtered through a 400-micron mesh sieve, and subsequently provided to the larvae. The quantity of *Daphnia* allocated for feeding *Daphnia*-fed larvae constituted 80% of the living biomass of the larvae per day [1]. While feeding *Daphnia* to treatments 3 and 4, frozen Chironomids were defrosted, finely chopped into the pieces smaller than the larvae's mouth using an electric shredding machine (Pars Khazar, Rasht, Iran), and introduced into the tanks to serve as feed for the larvae.

### 2.3. Fatty Acids Analysis

To assess the FA profile, 20 randomly selected fish at 11 days postfeeding from each tank were euthanatized using clove powder extract. The fish samples were carefully collected, placed in microtubes, and promptly frozen in liquid nitrogen. Subsequently, these samples were immediately transferred to the Aquatic Laboratory in the Faculty of Natural Resources, University of Guilan (Sowmeh Sara, Guilan, Iran), and stored at −80°C until analysis. The FA profiles of all live foods were also analyzed. The measurement of FA composition involved two steps: fat extraction and esterification [22, 23]. The fat extraction was carried out using the methanol–chloroform extraction method, followed by fat esterification using 2% methanolic sodium and BF_3_ (boron trifluoride). Subsequently, the FA samples were analyzed using a gas chromatograph (Philips, Sussex, England) equipped with a capillary column of SGE BPX70 (ID: 0.25 mm × 0.22 *µ*m × 30 m). The flame ionization detector operated at a temperature of 300°C, with the injector set at 250°C. A volume of 0.2 *μ*L of the ester sample was injected into the gas chromatograph for analysis. The column's initial temperature was set at 160°C, gradually increased to 230°C, and maintained this temperature for 5 min until all the compounds eluted. Helium served as the carrier gas, with hydrogen as the fuel, nitrogen as the auxiliary gas, and synthetic air in this method. By comparing the chromatograms' inhibition times of unknown samples with those obtained from standard solution, FAs in the fish body were identified, and the data were expressed as percentage. The FA profiles of the live foods used for larvae feeding were also measured.

### 2.4. Amino Acids Analysis

Larval sampling for AA analysis followed the same protocol as that for FA. To measure the AA profile, 0.2 g of fish sample was initially homogenized with 10 mL of 6 M sodium hydrochloric acid and placed in an oven at 110°C for 24 hr. Subsequently, the hydrolyzed samples were cooled to ambient temperature and filtered. The resulting solution (20 mL) was dried using nitrogen gas and then mixed with 100 *μ*L buffer containing sodium acetate, copper acetate, and hexane sulfate. After centrifugation, 20 *μ*L of the sample was injected into the HPLC (Perkin Elmer, Series 200, USA) using an isocratic method with a solvent at a rate of 0.7 mL/min, following the method outlined by Levin and Grushka [24]. The AA profiles of the live foods provided to the larvae were also measured.

The following equation was used to determine the relative difference between the composition of EAAs in larval samples and the consumed food to calculate the limiting EAA [25]:

The limiting EAA = 100 × (larval EAA − food EAA)/larval EAA.

### 2.5. Statistical Analysis

The data were subjected to analysis using SPSS version 25 (IBM SPSS Statistics, Armonk, USA). The normality of the data was assessed through the Kolmogorov–Smirnov test, and Leven's test was employed to evaluate the homogeneity of variances. One-way analysis of variance (ANOVA) was utilized to determine difference in the means profiles of FA and AA among the groups, while the independent-samples *t*-test was employed for comparison between two groups (larvae and live food). Tukey's test was applied to determine significant differences between means, with a significant level set at *P*  < 0.05. Additionally, Excel software (ver. 2019, Microsoft, USA) was used to determine the correlation coefficient between the EAA of the larvae and the AA of the live foods.

## 3. Results

### 3.1. Average Weight and Survival Rate

**Figure 1:**
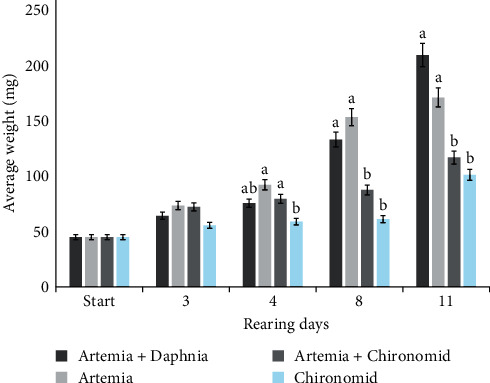
Mean weight of Persian sturgeon larvae during 11 days of rearing. Different letters on the columns showed significant difference among treatments in each time (*P*  < 0.05).

Figure 1 illustrates the trend in the average weight of larvae over the 11-day rearing period. On day 4, there was no significant difference in weight among the treatments. However, by the end of this period, treatment 1 (*Artemia* + *Daphnia*) showed the highest weight, while treatment 4 (Chironomid) showed the lowest weight (*P*  < 0.05). In terms of survival rate, no significant difference was observed among the treatments (*P*  > 0.05).

### 3.2. FA Profiles

Table 1 presents the results of the FA profile of the live foods utilized in the feeding of Persian sturgeon larvae. The highest percentage of EPA, DHA, and SFA was observed in *Daphnia* and the lowest in *Artemia* (*P*  < 0.05). Moreover, oleic (*ω*9) and DHA/EPA ratio were most abundant in *Daphnia*, contrasting with Chironomid where they were least abundant (*P*  < 0.05). The DHA + EPA ratio was highest in *Daphnia* and lowest in *Artemia*(*P*  > 0.05). *Artemia* exhibited the highest content of polyunsaturated fatty acids (PUFAs) and n3/n6 ratio, while Chironomid displayed the lowest values for these parameters (*P*  < 0.05). Additionally, *Artemia* had the highest percentage of *ω*3 and the lowest of *ω*6, whereas *Daphnia* showed the lowest *ω*3 and Chironomid had the highest *ω*6 (*P*  < 0.05). The fat contents of *Artemia* nauplii, Chironomid, and *Daphnia* were 13.9%, 13%, and 25.0%, respectively.

Table 2 presents the FA profile in the whole body of Persian sturgeon larvae. Treatment 1 (*Artemia* + *Daphnia*) exhibited the highest levels of EPA, DHA + EPA, omega 3, *ω*6, DHA/EPA, SFA, MUFA, and n3/n6 ratio (*P*  < 0.05). Treatment 2 (only *Artemia*) had the highest levels of DHA and oleic acid (*ω*9) (*P*  < 0.05). Treatment 3 had the highest percentage of PUFA (*ω*3 + *ω*6) (*P*  < 0.05).

Table 3 presents a comparison of the FA composition of Persian sturgeon larvae with that of the consumed live foods. In treatment 1, where larvae were fed *Artemia* and *Daphnia*, the larvae exhibited higher levels of EPA, DHA, DHA/EPA, and DHA + EPA compared to *Daphnia*, while showing lower levels of *ω*3, MUFA, and *n*-3/*n*-6 ratio than *Daphnia* (*P*  < 0.05). Moreover, larvae in treatment 1 showed higher amounts of EPA, DHA, *ω*3, DHA/EPA, and DHA + EPA, and lower amounts of *ω*9, *ω*3, *ω*6, and *n*-3/*n*-6 ratio compared to *Artemia*(*P*  < 0.05).

In treatment 2, where larvae were fed *Artemia*, the larvae exhibited higher levels of *ω*9, EPA, DHA, SFA, MUFA, DHA/EPA, and DHA + EPA and lower levels of *ω*3 and *n*-3/*n*-6 ratio compared to *Artemia* (*P*  < 0.05).

In treatment 3, where larvae were initially fed with *Artemia* for the first 3 days and subsequently with Chironomid for the next 8 days, the larvae exhibited higher levels of DHA, SFA, *ω*6, DHA/EPA, and DHA + EPA ratios, while showing lower levels of *ω*9, *ω*3, and *n*-3/*n*-6 ratio compared to *Artemia* (*P*  < 0.05). Additionally, in treatment 3, larvae displayed higher levels of *ω*9, *ω*3, DHA/EPA, and DHA + EPA and lower levels of SFA compared to Chironomid (*P*  < 0.05).

In treatment 4, where larvae were exclusively fed with Chironomid, the larvae showed higher level of *ω*9, DHA, DHA/EPA, DHA + EPA, and *n*-3/*n*-6 ratio compared to Chironomid, while exhibiting lower levels of SFA (*P*  < 0.05).

### 3.3. AA Profiles

Table 4 outlines the AA profiles of live foods used to feed Persian sturgeon larvae. *Artemia* exhibited the highest percentages of arginine, leucine, threonine, valine, isoleucine, and methionine (*P*  < 0.05). The highest percentages of lysine, phenylalanine, and EAAs were found in *Daphnia* (*P*  < 0.05). Histidine and nonessential amino acids (non-EAAs) were most abundant in Chironomid (*P*  < 0.05). The protein contents of *Artemia* nauplii, Chironomid, and *Daphnia* were 52.5%, 76%, and 39.7%, respectively.

Table 5 presents the AA profiles of Persian sturgeon larvae after 11 days of feeding with different live foods. Leucine in treatment 1 exhibited higher levels than the other treatments (*P*  < 0.05), while the levels of other AAs did not show significant differences among treatments (*P*  > 0.05).

Table 6 provides a comparison between the AA composition of the body of Persian sturgeon larvae and the live foods. In treatment 1, where Persian sturgeon larvae were fed with *Artemia* and *Daphnia*, leucine in larvae was higher than in *Daphnia*, while lysine in larvae was lower than in *Daphnia* (*P*  < 0.05). Moreover, non-EAAs in the larval body were higher than in *Artemia*, and arginine, histidine, isoleucine, methionine, serine, and total EAAs in larvae were lower than in *Daphnia* (*P*  < 0.05).

In treatment 2, where larvae were exclusively fed with *Artemia*, phenylalanine and non-EAAs in the larval body were higher than in *Artemia*. In addition, arginine, isoleucine, valine, methionine, serine, and EAAs were lower than in *Artemia*(*P*  < 0.05).

In treatment 3, where larvae were fed with *Artemia* and Chironomid, leucine, lysine and non-EAAs in the larval body were higher than in Chironomid, while serine and EAAs were lower than in Chironomid (*P*  < 0.05). Non-EAAs in the larval body were higher than in *Artemia*, and arginine, leucine, methionine, serine, and EAAs were lower than in *Artemia*.

In treatment 4, where larvae were exclusively fed with Chironomid, leucine and lysine in the larval body were higher than in Chironomid, while phenylalanine and serine were lower than in Chironomid (*P*  < 0.05).

**Figure 2:**
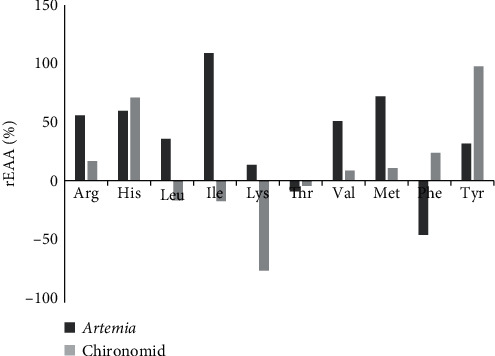
Relative proportions of different essential amino acids (relative essential amino acid % (rEAA %)) from the diet and the larvae which is calculated by the formula: rEAA (%) = 100 × (diet EAA − larval EAA)/larval EAA.

Figure 2 shows the relative differences (rEAA, %) between the EAA profiles of Persian sturgeon larvae (larval EAA) and those of live foods (live food EAA) after 11 days of feeding with *Artemia* in treatment 2 and Chironomid in treatment 4. As shown, *Artemia* is deficient in threonine and phenylalanine, while Chironomid is deficient in leucine, isoleucine, threonine, and lysine.

**Figure 3:**
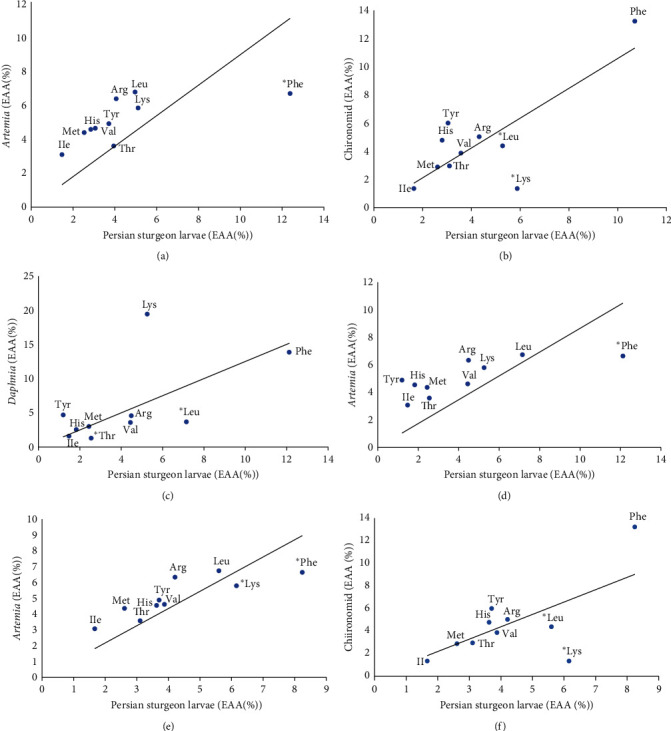
Comparison of the essential amino acid profiles (%) of Persian sturgeon (*A. persicus*) larvae, 11 days after feeding and the EAA profiles of (a) *Artemia* with larvae in treatment 2, (b) Chironomid with larvae in treatment 4, (c) *Daphnia* with larvae in treatment 1, (d) *Artemia* with larvae in treatment 1, (e) *Artemia* with larvae in treatment 3, and (f) Chironomid with larvae in treatment 3. The low points on the slope indicate a lack of AA in live food. AA profiles where the difference between larvae and live food is greatest are marked with an asterisk (*P*  < 0.05).

Figure 3 illustrates the correlation of EAA between Persian sturgeon larvae and live foods. In treatment 2 (Figure 3(a)), where the larvae were fed only *Artemia*, the correlation coefficient between larvae and *Artemia* compared to larvae fed only Chironomid (Figure 3(b)). The relationship between larval EAAs and live food EAAs in treatment 1, fed with *Artemia* and *Daphnia*, is depicted in Figure 3(c). The correlation coefficients between *Daphnia* with larvae and *Artemia* with larvae in this treatment were lower than those in treatment 2 (Figure 3(d)). The relationships between larval EAAs and *Artemia* EAAs, as well as Chironomid EAAs, are shown in Figures 3(e) and 3(f). The correlation coefficient between *Artemia* EAAs and larval EAAs in treatment 3 was higher than that in treatment 2 (fed with only *Artemia*), and also the correlation coefficient between Chironomid and larvae in treatment 3 was higher than in treatment 4 (fed with only Chironomid).

## 4. Discussion

In this study, the FA profiles of larvae fed with different live foods at the termination of the 11-day rearing period revealed distinct impacts of live foods on the FA profile of larvae across different treatments. Significant differences were observed in the profiles of larvae fed with *Artemia* + *Daphnia* and *Artemia* + Chironomid, as well as those exclusively fed with *Artemia* or Chironomid. These findings underscore the significant effect of live foods on the FA composition of Persian sturgeon larvae. The nutritional performance of fish larvae in utilizing live food is influenced by several factors, including the biochemical composition of the live food [25, 26]. The FA composition of larval body, when exposed to different live food sources, depends on both the profile of the live food and the fish's ability to digest and absorb these nutrients [12–27].

Numerous studies have highlighted that the FA composition of fish tissue is influenced by the dietary FA profile [28–30]. The findings of the present study, particularly in larvae exclusively fed with Chironomid, are consistent with the abovementioned studies, underscoring that the FA composition of larvae can be affected by the FA composition of their diet. Yoon et al. [30] used enriched *Artemia* and Chironomid on lake sturgeon larvae at an age older than the present research and found no significant difference in FA profile of the lavae. The difference in results may be due to the type of fish, age, and experimental condition.

In the current study, DHA/EPA and *n*-3/*n*-6 content varied in Persian sturgeon larvae fed with different foods. These ratios were higher in larvae fed with *Artemia* + *Daphnia* and those fed only with *Artemia* compared to the larvae fed with only Chironomid and *Artemia* + Chironomid. These results are consistent with the findings of Bae and Lim [31] and Všetičková [32], who suggested that EPA + DHA levels are influenced by genetics, nutrition, and environmental factors, such as salinity and season. In this study, all conditions were identical except for the type of feeding in the treatments.

In all treatments, EPA and DHA levels in the larval profile exceeded those in the live foods. This pattern is consistent with observations in other species such as white sturgeon (*Acipenser transmontanus*) [33], Russian sturgeon (*Acipenser gueldenstaedtii*) [34], Beluga (*Huso huso*) [35], Siberian sturgeon (*Acipenser baerii*) [36], and golden pompano (*Trachinotus ovatus*) larvae [37]. They found that levels of EPA and DHA in fish muscle surpassed those in the diet, indicating that UFAs are replaced in the fish body through elongation or nonelongation processes involving other FAs. Alpha-linolenic acid serves as an essential precursor for the elongation and unsaturation processes leading to the formation of DHA and EPA. Freshwater fish outperform the marine fish for unsaturation and elongation of FA to larger homologues [38, 39]. The increase in EPA and DHA levels in the larvae compared to different live foods across all treatments suggests that Persian sturgeon larvae at the early life stages possess the ability to effectively use EPA and DHA from live foods and store them in their body. This function appears to be particularly pronounced in larvae fed with Chironomid compared to other live foods.

The content of AA, especially EAA, is crucial for fish health and growth. The EAA composition in fish carcasses serves as an appropriate indicator to estimate and determine the nutritional requirements of fish [19–29, 31–40]. The content and composition of AA in fish body depends on factors, such as nutrition, age, and season [41, 42]. Since fish larvae are unable to synthesize EAA [43, 44], they must obtain EAA through exogenous feeding. Differences in the AA content of food can cause a change in the AA composition of the larvae [18–29, 31–44].

Babaei et al. [45] reported an increase in the amount of leucine, methionine, and arginine in the larval body after changing the diet from *Artemia* to *Daphnia* in Persian sturgeon. In the current study, although the type of live food did not significantly change the AA composition of the larval body, leucine in larvae fed with *Artemia* + *Daphnia* was found to be significantly higher than in other treatments.

Upon yolk sac absorption, larval survival and growth become dependent on the amount of dietary AA [32–46]. In the present study, the EAA composition in Persian sturgeon larvae differed from the EAA composition in live foods. Larvae fed with Chironomid indicated higher correlation coefficients in their dietary EAA profiles as compared to larvae fed with *Artemia*. This suggests that *Artemia* is less balanced in terms of EAA than Chironomid.

To show the effects of changing live food for larval feeding, the correlation coefficient between larval EAAs and live food EAAs (EAA larvae/EAA live food) in these treatments was evaluated and compared with treatment 2, where larvae were fed only *Artemia*. The results showed an increased correlation coefficient in treatment 3, where larvae were fed with *Artemia* and Chironomid, compared to treatment 2 with only *Artemia*. However, in treatment 1, where larvae were fed with *Artemia* and *Daphnia*, the correlation coefficient between larvae and *Daphnia* decreased compared to larvae fed only *Artemia*.

In order to achieve optimal nutritional balance in larval diets, it is necessary to determine the limiting EAAs [25]. Our results revealed variations in the EAAs profiles between of Persian sturgeon larvae and live foods. *Artemia* was deficient in threonine and phenylalanine for larval nutrition, with phenylalanine being the first limiting amino acid. Chironomid, on the other hand, exhibited deficiency in leucine, isoleucine, lysine, and threonine, with lysine being the first limiting amino acid.

In the case of feeding Persian sturgeon larvae with *Daphnia*, deficiencies in arginine, leucine, isoleucine, valine, threonine, and phenylalanine were observed, with phenylalanine identified as the first limiting AA. Comparable studies in turbot (*Scophthalmus maximus*) fed with *Artemia* reported deficiencies in threonine, methionine, and leucine [47]. Babaei et al. [45] reported that Persian sturgeon larvae fed with *Artemia* exhibited deficiencies in arginine, histidine, leucine, lysine, and methionine on the 14th day. After switching to *Daphnia*, deficiencies in histidine, lysine, and threonine were observed on the 40th day, with phenylalanine being the restrictive AA [45]. The observed differences in EAA deficiencies between studies may be attributed to changes in the EAA requirements of larvae at different ages and under varied experimental conditions. Consistent with the findings of Conceição et al. [47], our study, focusing on feeding of larvae with *Artemia*, identified a deficiency in threonine in the diet. Discrepancies in EAA deficiencies among studies could be attributed to variations in fish species.

In the present study, all foods used in larval regimes were deficient in EAA. Despite the differences in EAA content in live foods, our study did not reveal significant differences between treatments in terms of AA profile of Persian sturgeon larvae. The only exception was leucine, which was higher in treatments fed with *Artemia* and *Daphnia* compared to other treatments. Babaei et al. [45] also reported elevated levels of leucine in Persian sturgeon larvae fed with *Artemia* and *Daphnia* compared to other AAs.

In the present study, despite significant difference in AAs among live foods, only leucine exhibited a significant difference in treatment 1 compared to other treatments. A comparison between the AAs in larvae and live foods illustrated that some AAs in the larvae's body were more abundant than in live food, while others were less. The study highlighted the ability of Persian sturgeon larvae to balance EAAs in their bodies, as no significant differences were observed in the EAA profiles of larvae across treatments. The requirement for phenylalanine and tyrosine strongly increases in the metamorphic and early larval stages of fish [48]. The analysis of EAA profiles in larvae compared to EAAs in live food showed that *Artemia* was deficient in phenylalanine for feeding Persian sturgeon. This aligns with the principle that animals cannot synthesize the carbon chain of EAAs in their bodies and must obtain these EAAs through their diet [49, 50]. Interestingly, changing the type of live food did not significantly alter the EAA profile of Persian sturgeon larvae, possibly due to the absence of simultaneous feeding with both types of live food.

In general, it can be concluded that feeding Persian sturgeon larvae with different live foods, either alone or in combination, causes differences in body FA composition, while not causing significant differences in AA composition. Despite differences in AAs among live foods, Persian sturgeon larvae demonstrate an ability to establish a balanced state of AAs in their bodies. Additionally, the findings suggest that the larvae possess the ability to convert FAs from live foods into long-chain FAs, such as EPA and DHA, which would be more beneficial for both survival and growth.

The study results highlight that Persian sturgeon larvae fed with *Artemia* + *Daphnia* exhibited higher EPA + DHA content and a favorable *n*-3/*n*-6 ratio compared to other treatments. Although there was no significant difference in the AA profiles among larvae fed with different live foods, leucine content was notably higher in those feds with *Artemia* + *Daphnia*. Consequently, the larvae in the *Artemia* + *Daphnia* treatment showed superior performance in terms of AAs and UFA, contributing to a higher average weight at the end of the rearing period. This enhanced growth could be attributed to the larvae's preference for actively moving live foods like *Artemia* and *Daphnia* over less mobile options such as crushed Chironomid. Crushing Chironomid into fine particles may lead to the leaching of some nutrients into the water, rendering them inaccessible to the larvae. To address the phenylalanine deficiency in *Artemia*, enrichment before feeding is recommended. Overall, the study suggests that rearing larvae with *Artemia* + *Daphnia* at this stage is optimal for growth, with potential improvements through nutritional enrichment.

## Figures and Tables

**Table 1:** Fatty acids profile (%) of live foods used as feeds of the Persian sturgeon (*A. persicus*) larvae in the present experiment (mean ± SE; *n* = 3 replicates).

FA				Live food		
				Chironomid	*Artemia*	*Daphnia*
SFA		Lauric acid	C12 : 0	0.31 ± 0.06^b^	0.00 ± 0.00^b^	6.63 ± 0.10^a^
		Myristic acid	C14 : 0	5.32 ± 0.10^b^	1.19 ± 0.10^c^	7.44 ± 0.10^a^
		Pentadecanoic acid	C15 : 0	1.95 ± 0.09^a^	0.23 ± 0.10^b^	0.00 ± 0.00^b^
		Palmitic acid	C16 : 0	19.89 ± 0.19^a^	15.63 ± 0.12^b^	15.92 ± 0.10^b^
		Heptadecanoic	C17 : 0	3.05 ± 0.06^a^	2.37 ± 0.13^b^	0.60 ± 0.09^c^
		Stearic acid	C18 : 0	8.30 ± 0.13^c^	7.52 ± 0.11^b^	9.23 ± 0.09^a^
		Arachidic acid	C20 : 0	1.08 ± 0.10^a^	0.00 ± 0.00^b^	0.00 ± 0.00^b^

MUFA		Myristoleic acid	C14 : 1	4.85 ± 0.13^a^	1.74 ± 0.10^b^	0.00 ± 0.00^c^
		Pentadecenoic acid	C15 : 1	1.07 ± 0.09^a^	0.41 ± 0.08^b^	0.00 ± 0.10^b^
		Palmitoleic acid	C16 : 1	9.40 ± 0.17^a^	4.59 ± 0.10^b^	4.11 ± 0.10^b^
		Heptadecenoic acid	C17 : 1	2.85 ± 0.10^a^	2.30 ± 0.08^a^	0.35 ± 0.10^b^
		Oleic acid (*ω*9)	C18 : 1n9	18.48 ± 0.15^c^	26.13 ± 0.10^b^	32.37 ± 0.11^a^
		Eicosenoic acid	C20 : 1	0.60 ± 0.11^b^	2.13 ± 0.10^a^	0.60 ± 0.11^b^
		Docosenoic acid	C22 : 1	0.00 ± 0.00	0.23 ± 0.10	0.00 ± 0.00
PUFA	*ω*3	Linolenic acid	C18 : 3n3	3.34 ± 0.12^b^	23.32 ± 0.12^a^	2.79 ± 0.12^b^
		Eicosatrienoic acid	C20 : 3n3	0.00 ± 0.00	0.21 ± 0.07	0.00 ± 0.00
		Eicosapentaenoic acid (EPA)	C20 : 5n3	0.62 ± 0.11^b^	0.37 ± 0.08^b^	2.58 ± 0.12^a^
		Docosapentaenoic acid	C22 : 5n3	0.00 ± 0.00	0 ± 0.00	0.30 ± 0.11
		Docosahexaenoic acid (DHA)	C22 : 6n3	0.06 ± 0.01^b^	0.04 ± 0.10^b^	1.62 ± 0.12^a^
	*ω*6	Linoleic acid	C18 : 2n6	17.17 ± 0.14^a^	9.78 ± 0.10^c^	12.98 ± 0.14^b^
		*γ*-Linoleic acid	C18 : 3n6	0.68 ± 0.09^a^	0.43 ± 0.09^ab^	0.00 ± 0.00^b^
		Octadecatrienoic acid	C20 : 3n6	0.00 ± 0.00	0.00 ± 0.00	0.00 ± 0.00
		Arachionic acid	C20 : 4n6	1.00 ± 0.09^b^	1.04 ± 0.10^b^	2.96 ± 0.11^a^
		Adrenic acid	C22 : 4n6	0.00 ± 0.00	0.26 ± 0.13	0.00 ± 0.00

Total		SFA		39.9 ± 0.33^a^	26.94 ± 0.11^b^	39.82 ± 0.10^a^
		MUFA		37.24 ± 0.01	37.53 ± 0.08	37.43 ± 0.21
		*ω*3		4.02 ± 0.23^c^	23.94 ± 0.30^a^	7.30 ± 0.26^b^
		*ω*6		18.85 ± 0.32^a^	11.51 ± 0.17^c^	15.95 ± 0.03^b^
		*ω*3 + *ω*6		22.87 ± 7.13^b^	35.45 ± 0.13^a^	23.24 ± 0.23^b^
		DHA/EPA		0.09 ± 0.01^b^	0.11 ± 0.02^b^	0.61 ± 0.03^a^
		DHA + EPA		0.68 ± 0.11^b^	0.41 ± 0.10^b^	4.26 ± 0.18^a^
		*n*-3/*n*-6		0.21 ± 0.02^c^	2.08 ± 0.06^a^	0.46 ± 0.02^b^

Dissimilar letters in each row indicate a significant difference among the treatments (*P*  < 0.05).

**Table 2:** Fatty acids profile (%) of whole body of Persian sturgeon (*A. persicus*) larvae after 11 days feeding with different live foods (mean ± SE; *n* = 3).

FA				Treatments			
				*Artemia* + *Daphnia*	*Artemia*	*Artemia* + Chironomid	Chironomid
SFA		Lauric	C12 : 0	0.00 ± 0.00	0.00 ± 0.00	0.25 ± 0.02	0.61 ± 0.02
		Myristic	C14 : 0	1.22 ± 0.35^b^	1.01 ± 0.33^b^	2.78 ± 0.15^a^	3.26 ± 0.51^a^
		Pentadecanoic	C15 : 0	0.67 ± 0.09^b^	0.36 ± 0.15^b^	1.28 ± 0.07^a^	1.23 ± 0.05^a^
		Palmitic	C16 : 0	24.12 ± 0.88	21.65 ± 1.93	18.95 ± 0.28	18.86 ± 0.49
		Heptadecanoic	C17 : 0	1.89 ± 1.05	1.06 ± 0.39	2.68 ± 0.16	2.72 ± 0.05
		Stearic	C18 : 0	12.17 ± 0.45^a^	7.50 ± 0.69^b^	9.93 ± 0.45^ab^	8.98 ± 0.28^b^

MUFA		Myristoleic	C14 : 1	0.24 ± 0.04^b^	0.44 ± 0.33^ab^	1.68 ± 0.15^ab^	2.20 ± 0.51^a^
		Pentadecenoic	C15 : 1	0.45 ± 0.04^b^	0.12 ± 0.05^b^	0.51 ± 0.02^a^	0.61 ± 0.07^a^
		Palmitoleic	C16 : 1	5.62 ± 1.56	7.19 ± 0.93	8.35 ± 0.03	9.11 ± 1.10
		Heptadecenoic	C17 : 1	1.46 ± 0.02	1.29 ± 0.68	3.22 ± 0.14	3.15 ± 0.49
		Eicosenoic	C20 : 1	1.01 ± 0.71	1.18 ± 0.84	1.26 ± 0.89	1.27 ± 0.90
		Docosenoic	C22 : 1	0.30 ± 0.71	0.28 ± 0.83	024 ± 0.89	0.26 ± 0.90
		Oleic (*ω*9)	C18 : 1n9	28.50 ± 0.97^b^	35.37 ± 0.40^a^	22.72 ± 0.56^c^	22.75 ± 0.10^c^
UFA	*ω*3	Linolenic	C18 : 3n3	1.39 ± 0.05	1.40 ± 0.05	1.60 ± 0.04	1.35 ± 0.13
		Eicosatrienoic	C20 : 3n3	0.08 ± 0.08^b^	0.29 ± 0.01^ab^	0.42 ± 0.06^a^	0.44 ± 0.04^a^
		Eicosapentaenoic (EPA)	C20 : 5n3	3.85 ± 0.30^a^	2.46 ± 0.02^b^	0.84 ± 0.02^c^	0.89 ± 0.13^c^
		Docosapentaenoic	C22 : 5n3	1.72 ± 0.01^a^	0.90 ± 0.08^b^	0.63 ± 0.02^ab^	0.57 ± 0.08^c^
		Docosahexaenoic (DHA)	C22 : 6n3	5.84 ± 0.37^ab^	6.75 ± 0.85^a^	3.24 ± 0.02^b^	3.41 ± 0.63^b^
	*ω*6	Linoleic	C18 : 2n6	4.41 ± 1.42^b^	6.76 ± 1.27^ab^	11.33 ± 0.66^a^	10.72 ± 0.45^a^
		*γ*-Linoleic	C18 : 3n6	0.00 ± 0.00^b^	0.53 ± 0.12^b^	1.54 ± 0.11^a^	1.85 ± 0.14^a^
		Octadecatrienoic	C20 : 3n6	0.24 ± 0.06^b^	0.31 ± 0.06^b^	0.91 ± 0.12^a^	0.85 ± 0.00^a^
		Arachionic	C20 : 4n6	5.08 ± 0.39^a^	2.96 ± 0.38^b^	4.76 ± 0.45^ab^	4.47 ± 0.18^ab^
		Adrenic	C22 : 5n6	0.17 ± 0.04^b^	0.21 ± 0.00^b^	0.88 ± 0.07^a^	0.82 ± 0.13^a^
		Docosatetraenoic	C22 : 6n6	0.00 ± 0.00	0.00 ± 0.00	0.00 ± 0.00	0.02 ± 0.02

Total		SFA		40.07 ± 0.85^a^	31.58 ± 0.58^d^	35.63 ± 1.38^b^	35.11 ± 1.35^c^
		MUFA		37.17 ± 0.29^b^	45.87 ± 1.80^a^	37.97 ± 0.89^ab^	39.36 ± 1.90^ab^
		*ω*3		12.87 ± 0.43^a^	11.80 ± 0.49^b^	6.74 ± 0.22^c^	6.65 ± 0.24^c^
		*ω*6		9.90 ± 0.48^d^	10.75 ± 0.52^c^	19.42 ± 0.82^a^	18.73 ± 0.77^b^
		*ω*3 + *ω*6		22.67 ± 0.46^c^	22.55 ± 0.50^c^	26.16 ± 0.64^a^	25.38 ± 0.61^b^
		DHA/EPA		1.52 ± 0.02^b^	2.75 ± 0.37^a^	3.84 ± 0.11^a^	3.82 ± 0.13^a^
		DHA + EPA		9.69 ± 0.67^a^	9.21 ± 0.83^a^	4.09 ± 0.01^b^	4.30 ± 0.13^b^
		*n*-3/*n*-6		1.30 ± 0.30^a^	1.10 ± 0.10^b^	0.35 ± 0.00^c^	0.36 ± 0.01^c^

Dissimilar letters in each row indicate a significant difference among the treatments (*P*  < 0.05).

**Table 3:** Comparison of fatty acids of live foods and Persian sturgeon (*A. persicus*) larvae, 11 days after feeding with different live foods (mean ± SE; *n* = 3).

	Treatment 1	Live food		Treatment 2	Live food	Treatment 3	Live food		Treatment 4	Live food
	(*Artemia* + *Daphnia*)	*Artemia*	*Daphnia*	(Artemia)	*Artemia*	(*Artemia* + Chironomid)	*Artemia*	Chironomid	(Chironomid)	Chironomid
C12 : 0	0.00 ± 0.00^b^	0.00 ± 0.00^b^	6.63 ± 0.10^a^	0.00 ± 0.00	0.00 ± 0.00	0.25 ± 0.02	0.00 ± 0.00	0.31 ± 0.06	0.61 ± 0.02	0.31 ± 0.06
C14 : 0	1.22 ± 0.35^b^	1.19 ± 0.10^b^	7.44 ± 0.10^a^	1.01 ± 0.33	1.19 ± 0.10	2.78 ± 0.15^b^	1.19 ± 0.10^c^	5.32 ± 0.10^a^	3.26 ± 0.51	5.32 ± 0.10
C15 : 0	0.67 ± 0.09^a^	0.23 ± 0.10^ab^	0.00 ± 0.00^b^	0.36 ± 0.15	0.23 ± 0.10	1.28 ± 0.07^b^	0.23 ± 0.10^c^	1.95 ± 0.09^a^	1.23 ± 0.05	1.95 ± 0.09
C16 : 0	24.12 ± 0.88^a^	15.63 ± 0.12^b^	15.92 ± 0.10^b^	21.65 ± 1.93	15.63 ± 0.12	18.95 ± 0.28^a^	15.63 ± 0.12^b^	19.89 ± 0.19^a^	18.86 ± 0.49	19.89 ± 0.19
C17 : 0	1.89 ± 1.05	2.37 ± 0.13	0.60 ± 0.09	1.06 ± 0.39	2.37 ± 0.13	2.68 ± 0.16	2.37 ± 0.13	3.05 ± 0.06	2.72 ± 0.05	3.05 ± 0.06
C18 : 0	12.17 ± 0.45^a^	7.52 ± 0.11^c^	9.23 ± 0.09^b^	7.50 ± 0.69	7.52 ± 0.11	9.93 ± 0.45^a^	7.52 ± 0.11^b^	8.30 ± 0.13^b^	8.98 ± 0.28	8.30 ± 0.13
C20 : 0	0.00 ± 0.00	0.00 ± 0.00	0.00 ± 0.00	0.00 ± 0.00	0.00 ± 0.00	0.00 ± 0.00^b^	0.00 ± 0.00^b^	1.08 ± 0.10^a^	0.00 ± 0.00^b^	1.08 ± 0.10^a^
C14 : 1	0.24 ± 0.04^b^	1.74 ± 0.10^a^	0.00 ± 0.00^b^	0.44 ± 0.23	1.74 ± 0.10	1.68 ± 015^b^	1.74 ± 0.10^b^	4.84 ± 0.13^a^	2.20 ± 0.73	4.85 ± 0.13
C15 : 1	0.05 ± 0.04^b^	0.41 ± 0.08^a^	0.00 ± 0.00^b^	0.12 ± 0.05	0.41 ± 0.08	0.51 ± 0.02^b^	0.41 ± 0.08^b^	1.07 ± 0.09^a^	0.61 ± 0.07	1.07 ± 0.09
C16 : 1	5.62 ± 1.56	4.59 ± 0.10	4.11 ± 0.10	7.19 ± 0.93	4.59 ± 0.10	8.35 ± 0.03^b^	4.59 ± 0.10^c^	9.40 ± 0.17^a^	9.11 ± 1.10	9.40 ± 0.17
C17 : 1	1.46 ± 0.02^b^	2.30 ± 0.08^a^	0.35 ± 0.10^c^	1.29 ± 0.68^b^	2.30 ± 0.08^a^	3.22 ± 0.14^a^	2.30 ± 0.08^b^	2.85 ± 0.10^ab^	3.15 ± 0.49	2.85 ± 0.10
C18 : 1n9	28.50 ± 0.97^b^	26.13 ± 0.10^b^	32.37 ± 0.11^a^	35.37 ± 0.40^a^	26.13 ± 0.10^b^	22.72 ± 0.56^b^	26.13 ± 0.10^a^	18.48 ± 0.15^c^	22.75 ± 0.10^a^	18.48 ± 0.15^b^
C20 : 1	1.01 ± 0.71^b^	2.13 ± 0.10^a^	0.60 ± 0.11^b^	1.18 ± 0.84^b^	2.13 ± 0.10^a^	1.26 ± 0.89^ab^	2.13 ± 0.10^a^	0.60 ± 0.11^b^	1.27 ± 0.90	0.60 ± 0.11
C18 : 3n3	1.39 ± 0.05^c^	23.32 ± 0.12^a^	2.79 ± 0.12^b^	1.40 ± 0.05^b^	23.32 ± 0.12^a^	1.60 ± 0.04^c^	23.32 ± 0.12^a^	3.34 ± 0.12^b^	1.35 ± 0.13^b^	3.34 ± 0.12^a^
C20 : 3n3	0.08 ± 0.08	0.21 ± 0.07	0.00 ± 0.00	0.29 ± 0.01	0.21 ± 0.07	0.42 ± 0.06^a^	0.21 ± 0.07^ab^	0.00 ± 0.00^b^	0.44 ± 0.04^a^	0.00 ± 0.00^b^
C20 : 5n3	3.85 ± 0.30^a^	0.37 ± 0.08^c^	2.58 ± 0.12^b^	2.46 ± 0.02^a^	0.37 ± 0.08^b^	0.84 ± 0.02	0.37 ± 0.08	0.62 ± 0.11	0.89 ± 0.13	0.62 ± 0.11
C22 : 5n3	1.72 ± 0.01^a^	0.00 ± 0.00^b^	0.30 ± 0.11^b^	4.21 ± 0.08^a^	0.00 ± 0.00^b^	0.63 ± 0.02^a^	0.00 ± 0.00^b^	0.00 ± 0.00^b^	0.57 ± 0.08	0.00 ± 0.00
C22 : 6n3	5.84 ± 0.37^a^	0.04 ± 0.10^c^	1.62 ± 0.12^b^	6.75 ± 0.85^a^	0.04 ± 0.10^b^	3.24 ± 0.02^a^	0.04 ± 0.10^b^	0.06 ± 0.01^b^	3.41 ± 0.63^a^	0.06 ± 0.01^b^
C18 : 2n6	4.41 ± 1.42^b^	9.78 ± 0.10^a^	12.98 ± 0.14^a^	6.76 ± 1.27^b^	9.78 ± 0.10^a^	11.33 ± 0.66^b^	9.78 ± 0.10^b^	17.17 ± 0.14^a^	10.72 ± 0.45	17.17 ± 0.14
C18 : 3n6	0.00 ± 0.00^b^	0.43 ± 0.09^a^	0.00 ± 0.00^b^	0.53 ± 0.12	0.43 ± 0.09	1.54 ± 0.11^a^	0.43 ± 0.09^b^	0.68 ± 0.09^b^	1.85 ± 0.14	0.68 ± 0.09
C20 : 3n6	0.24 ± 0.06^a^	0.00 ± 0.00^b^	0.00 ± 0.00^b^	0.31 ± 0.06^a^	0.00 ± 0.00^b^	0.91 ± 0.12^a^	0.00 ± 0.00^b^	0.00 ± 0.00^b^	0.85 ± 0.00^a^	0.00 ± 0.00^b^
C20 : 4n6	5.08 ± 0.39^a^	1.04 ± 0.10^c^	2.96 ± 0.11^b^	2.96 ± 0.38	1.04 ± 0.10	4.76 ± 0.45^a^	1.04 ± 0.10^b^	1.00 ± 0.09^b^	4.47 ± 0.18	1.00 ± 0.09
C22 : 4n6	0.17 ± 0.04	0.26 ± 0.15	0.00 ± 0.00	0.21 ± 0.00	0.26 ± 0.15	0.88 ± 0.07^a^	0.26 ± 0.15^b^	0.00 ± 0.00^b^	0.82 ± 013^a^	0.00 ± 0.00^b^
C22 : 5n6	0.26 ± 0.15	0.00 ± 0.00	0.00 ± 0.00	0.21 ± 0.00^a^	0.00 ± 0.00^b^	0.00 ± 0.00	0.00 ± 0.00	0.00 ± 0.00	0.15 ± 0.00^a^	0.00 ± 0.00^b^
C22 : 6n6	0.00 ± 0.00	0.00 ± 0.00	0.00 ± 0.00	0.00 ± 0.00	0.00 ± 0.00	0.00 ± 0.00	0.00 ± 0.00	0.00 ± 0.00	0.00 ± 0.00	0.00 ± 0.00
SFA	40.07 ± 0.85^a^	26.94 ± 0.11^b^	39.82 ± 0.10^a^	31.58 ± 0.58^a^	26.94 ± 0.11^b^	35.63 ± 1.38^b^	26.94 ± 0.11^c^	39.89 ± 0.10	35.11 ± 1.35^b^	39.89 ± 0.33^a^
MUFA	37.17 ± 0.29	37.54 ± 0.08	37.43 ± 0.21	45.87 ± 1.80^a^	37.54 ± 0.08^b^	37.97 ± 0.89	37.54 ± 0.08	37.24 ± 0.33	39.36 ± 1.90	37.24 ± 0.01
*ω*3	12.87 ± 0.43^b^	24.23 ± 0.30^a^	7.03 ± 0.26^c^	11.80 ± 0.49^b^	23.94 ± 0.30^a^	6.72 ± 0.22^b^	23.94 ± 0.30^a^	4.02 ± 0.01^c^	6.65 ± 0.24^a^	4.02 ± 0.23^b^
*ω*6	9.90 ± 0.48^c^	11.54 ± 0.17^b^	15.95 ± 0.03^a^	10.70 ± 0.52	11.51 ± 0.17	19.42 ± 0.82^a^	11.51 ± 0.17^b^	18.85 ± 0.32^a^	18.73 ± 0.77	18.85 ± 0.32
(*ω*3 + *ω*6)	22.77 ± 0.46^b^	35.54 ± 0.13^a^	23.24 ± 0.23^b^	22.55 ± 0.50^b^	35.45 ± 0.13^a^	26.14 ± 0.64^b^	35.45 ± 0.13^a^	22.87 ± 7.13^b^	25.38 ± 0.61^b^	30.24 ± 7.13^a^
DHA/EPA	1.52 ± 0.02^a^	0.11 ± 0.02^c^	0.61 ± 0.03^b^	2.75 ± 0.37^a^	0.11 ± 0.02^b^	3.84 ± 0.11^a^	0.11 ± 0.02^b^	0.09 ± 0.01^b^	4.30 ± 0.13^a^	0.68 ± 0.01^b^
DHA + EPA	9.69 ± 0.67^a^	0.41 ± 0.10^c^	4.26 ± 0.18^b^	9.21 ± 0.83^a^	0.41 ± 0.10^b^	4.09 ± 0.01^a^	0.41 ± 0.10^b^	0.67 ± 0.11^b^	3.82 ± 0.13^a^	0.89 ± 0.11^b^
*n*-3/*n*-6	1.30 ± 0.30^b^	2.08 ± 0.06^a^	0.46 ± 0.02^c^	1.11 ± 0.10^b^	2.08 ± 0.06^a^	0.35 ± 0.00^b^	2.08 ± 0.06^a^	0.21 ± 0.02^b^	0.36 ± 0.01^a^	0.21 ± 0.02^b^

Dissimilar letters in each row indicate a significant difference among the treatments (*P*  < 0.05).

**Table 4:** Amino acids profile (%) of live foods used to feed the Persian sturgeon (*A. persicus*) larvae (mean ± SE; *n* = 3).

AA		Chironomid	*Artemia*	*Daphnia*
EAA	Arginine	5.03 ± 0.10^b^	6.34 ± 0.16^a^	4.59 ± 0.11^b^
	Histidine	4.79 ± 0.24^a^	4.55 ± 0.14^a^	2.56 ± 0.15^b^
	Leucine	4.39 ± 0.06^b^	6.64 ± 0.10^a^	3.69 ± 0.23^b^
	Isoleucine	1.36 ± 0.11^b^	3.07 ± 0.11^a^	1.60 ± 0.23^b^
	Lysine	1.35 ± 0.09^c^	5.79 ± 0.10^b^	19.45 ± 0.15^a^
	Threonine	2.96 ± 0.12^a^	3.58 ± 0.12^a^	1.31 ± 0.23^b^
	Valine	3.67 ± 0.11^ab^	4.62 ± 0.13^a^	3.60 ± 0.21^b^
	Methionine	2.88 ± 0.08^b^	4.36 ± 0.10^a^	3.02 ± 0.17^b^
	Phenylalanine	13.23 ± 0.05^a^	6.64 ± 0.14^b^	13.88 ± 0.46^a^
	Tyrosine	6.01 ± 0.09^a^	4.89 ± 0.12^b^	4.70 ± 0.21^b^

Non-EAA	Aspartic acid	9.09 ± 0.03^a^	8.28 ± 0.11^a^	6.79 ± 0.12^b^
	Glutamine	18.24 ± 0.06^a^	9.88 ± 0.08^c^	12.98 ± 0.22^b^
	Serine	7.28 ± 0.07^a^	7.16 ± 0.14^a^	4.91 ± 0.14^b^
	Glycine	6.55 ± 0.21^b^	10.80 ± 0.10^a^	5.47 ± 0.28^b^
	Alanine	6.29 ± 0.26^a^	6.02 ± 0.13^b^	5.08 ± 0.20^ab^
	Proline	5.49 ± 0.08^b^	7.29 ± 0.12^a^	4.18 ± 0.21^c^
	Hydroxyproline	1.21 ± 0.09^b^	0.00 ± 0.00^c^	2.21 ± 0.26^a^

Total	EAA	45.87 ± 0.25^c^	50.57 ± 0.40^b^	58.39 ± 0.25^a^
	Non-EAA	54.13 ± 0.25^a^	49.43 ± 0.40^b^	41.61 ± 0.24^c^

Dissimilar letters in each row indicate a significant difference among the treatments (*P*  < 0.05).

**Table 5:** Amino acids profile (%) of Persian sturgeon (*A. persicus*) larvae after 11 days of feeding with different live foods (mean ± SE; *n* = 3).

		Treatments			
		*Artemia* + *Daphnia*	*Artemia*	*Artemia* + Chironomid	Chironomid
EAA	Histidine	1.83 ± 0.03	2.85 ± 0.27	3.63 ± 0.13	2.80 ± 0.78
	Leucine	7.15 ± 0.28^a^	4.97 ± 0.23^b^	5.60 ± 0.20^b^	5.28 ± 0.12^b^
	Isoleucine	1.48 ± 0.47	1.47 ± 0.00	1.67 ± 0.01	1.64 ± 0.09
	Lysine	5.26 ± 0.59	5.11 ± 0.54	6.16 ± 0.08	5.88 ± 0.66
	Threonine	2.55 ± 0.47	3.94 ± 0.20	3.10 ± 0.27	3.10 ± 0.13
	Valine	4.44 ± 0.39	3.06 ± 0.49	3.87 ± 0.24	3.57 ± 0.01
	Methionine	2.44 ± 0.53	2.54 ± 0.02	2.61 ± 0.08	2.61 ± 0.25
	Phenylalanine	12.12 ± 2.60	12.38 ± 0.99	8.24 ± 0.38	10.70 ± 0.00
	Tyrosine	1.20 ± 0.10	3.71 ± 0.18	3.71 ± 0.66	3.04 ± 1.61

Non-EAA	Aspartic acid	8.85 ± 0.17	8.79 ± 0.39	8.12 ± 0.74	8.13 ± 0.95
	Glutamine	20.91 ± 0.28	18.17 ± 0.56	20.66 ± 0.12	20.61 ± 0.82
	Serine	4.33 ± 0.15	5.06 ± 0.15	6.83 ± 0.18	6.79 ± 0.38
	Glycine	8.49 ± 0.44	9.49 ± 0.27	8.98 ± 0.01	8.24 ± 0.12
	Alanine	6.74 ± 0.20	5.25 ± 0.35	5.92 ± 0.02	5.62 ± 0.38
	Proline	5.03 ± 0.17	5.23 ± 0.16	5.05 ± 0.18	5.71 ± 0.38
	Hydroxyproline	2.71 ± 0.32	3.91 ± 0.03	3.47 ± 0.10	3.07 ± 0.24

Total	EAA	42.93 ± 1.09	44.10 ± 0.09	42.80 ± 0.36	42.95 ± 2.78
	Non-EAA	57.07 ± 1.09	55.90 ± 0.09	57.20 ± 0.36	57.05 ± 2.78

Dissimilar letters in each row indicate a significant difference between different treatments (*P*  < 0.05).

**Table 6:** Comparison of amino acids of Persian sturgeon larvae and live foods after 11 days of feeding (mean ± SE; *n* = 3).

	Treatment 1	Live food		Treatment 2	Live food	Treatment 3	Live food		Treatment 4	Live food
	(*Artemia* + *Daphnia*)	*Artemia*	Daphnia	(Artemia)	*Artemia*	(*Artemia* + Chironomid)	*Artemia*	Chironomid	(Chironomid)	Chironomid
Arginine	4.48 ± 0.25^b^	6.34 ± 0.16^a^	4.59 ± 0.11^b^	4.07 ± 0.47^b^	6.34 ± 0.16^a^	4.21 ± 0.07^c^	6.34 ± 0.16^a^	5.03 ± 0.10^b^	4.32 ± 0.15	5.03 ± 0.10
Histidine	1.83 ± 0.03^c^	4.55 ± 0.14^a^	2.56 ± 0.15^b^	2.85 ± 0.27^b^	4.55 ± 0.14^a^	3.63 ± 0.13^b^	4.55 ± 0.14^ab^	4.78 ± 0.24^a^	2.80 ± 0.78	4.78 ± 0.24
Leucine	7.15 ± 0.28^a^	6.74 ± 0.10^a^	3.69 ± 0.23^b^	4.97 ± 0.23^b^	6.74 ± 0.10^a^	5.60 ± 0.20^b^	6.74 ± 0.10^a^	4.39 ± 0.06^c^	5.28 ± 0.12^a^	4.39 ± 0.06^b^
Isoleucine	1.48 ± 0.47	3.07 ± 0.11	1.60 ± 0.23	1.47 ± 0.00^b^	3.07 ± 0.11^a^	1.67 ± 0.01^b^	3.07 ± 0.11^a^	1.35 ± 0.11^b^	1.64 ± 0.09	1.35 ± 0.11
Lysine	5.26 ± 0.59^b^	5.79 ± 0.10^b^	19.45 ± 0.15^a^	5.11 ± 0.54	5.79 ± 0.10	6.16 ± 0.08^a^	5.79 ± 0.10^a^	1.35 ± 0.09^b^	5.88 ± 0.66^a^	1.35 ± 0.09^b^
Threonine	2.55 ± 0.47^ab^	3.58 ± 0.12^a^	1.31 ± 0.23^b^	3.94 ± 0.20	3.58 ± 0.12	3.10 ± 0.27	3.58 ± 0.12	2.96 ± 0.12	3.10 ± 0.13	2.96 ± 0.12
Valine	4.44 ± 0.39	4.62 ± 0.13	3.60 ± 0.21	3.06 ± 0.49	4.62 ± 0.13	3.87 ± 0.24	4.62 ± 0.13	3.87 ± 0.11	3.57 ± 0.01	3.87 ± 0.11
Methionine	2.44 ± 0.53	4.36 ± 0.10	3.02 ± 0.17	2.54 ± 0.02^b^	4.36 ± 0.10^a^	2.61 ± 0.08^b^	4.36 ± 0.10^a^	2.89 ± 0.08^b^	2.61 ± 0.25	2.89 ± 0.08
Phenylalanine	12.12 ± 2.60	6.64 ± 0.14	13.88 ± 0.46	12.38 ± 0.99^a^	6.64 ± 0.14^b^	8.24 ± 0.38^b^	6.64 ± 0.14^c^	13.23 ± 0.05^a^	10.70 ± 0.00^b^	13.23 ± 0.05^a^
Tyrosine	1.20 ± 0.10^b^	4.89 ± 0.12^a^	4.70 ± 0.21^a^	3.71 ± 0.18^b^	4.89 ± 0.12^a^	3.71 ± 0.66^b^	4.89 ± 0.12^ab^	6.01 ± 0.09^a^	3.04 ± 1.61	6.01 ± 0.09

Aspartic acid	8.85 ± 0.17^a^	8.28 ± 0.11^a^	6.79 ± 0.12^b^	8.79 ± 0.39	8.28 ± 0.11	8.12 ± 0.74	8.55 ± 0.11	9.09 ± 0.03	8.13 ± 0.95	9.09 ± 0.03
Glutamine	20.91 ± 0.28^a^	9.88 ± 0.08^c^	12.98 ± 0.22^b^	18.17 ± 0.56^a^	9.88 ± 0.08^b^	20.66 ± 0.12^a^	9.88 ± 0.08^c^	18.24 ± 0.06^b^	20.61 ± 0.82	18.24 ± 0.06
Serine	4.33 ± 0.15^b^	7.16 ± 0.14^a^	4.91 ± 0.14^b^	5.06 ± 0.15^b^	7.16 ± 0.14^a^	5.01 ± 0.18^b^	7.16 ± 0.14^a^	7.27 ± 0.07^a^	6.79 ± 0.38	7.27 ± 0.07
Glycine	8.49 ± 0.44^b^	10.80 ± 0.10^a^	5.46 ± 0.28^c^	9.49 ± 0.27^b^	10.80 ± 0.10^a^	8.98 ± 0.01^b^	10.80 ± 0.10^a^	6.55 ± 0.21^c^	8.24 ± 0.12^a^	6.55 ± 0.21^b^
Alanine	6.74 ± 0.20^a^	6.02 ± 0.13^ab^	5.08 ± 0.20^b^	5.25 ± 0.35	6.02 ± 0.13	5.92 ± 0.02	6.02 ± 0.13	6.28 ± 0.26	5.62 ± 0.38	6.28 ± 0.26
Proline	5.03 ± 0.17^b^	7.29 ± 0.12^a^	4.18 ± 0.21^b^	5.23 ± 0.16^b^	7.29 ± 0.12^a^	5.05 ± 0.18^b^	7.29 ± 0.12^a^	5.49 ± 0.08^b^	5.71 ± 0.38	5.49 ± 0.08
Hydroxyproline	2.71 ± 0.32^a^	0.00 ± 0.00^b^	2.21 ± 0.26^a^	3.91 ± 0.03^a^	0.00 ± 0.00^b^	3.47 ± 0.10^a^	0.00 ± 0.00^c^	1.21 ± 0.09^b^	3.07 ± 0.24^a^	1.21 ± 0.09^b^

EAA	42.93 ± 1.09^c^	50.57 ± 0.40^b^	58.39 ± 0.25^a^	44.10 ± 0.09^b^	50.57 ± 0.40^a^	42.80 ± 0.36^c^	50.57 ± 0.40^a^	45.87 ± 0.00^b^	42.95 ± 2.78	45.87 ± 0.00
Nonessential	57.07 ± 1.09^a^	49.43 ± 0.40^b^	41.61 ± 0.21^c^	55.90 ± 0.09^a^	49.43 ± 0.40^b^	57.20 ± 0.36^a^	49.43 ± 0.40^c^	54.13 ± 0.00^b^	57.05 ± 2.78	54.13 ± 0.00

Dissimilar letters in each row indicate a significant difference between different treatments (*P*  < 0.05).

## Data Availability

Data supporting this reasearch article are available on request.
